# Differences in gene mutations according to gender among patients with colorectal cancer

**DOI:** 10.1186/s12957-018-1431-5

**Published:** 2018-07-05

**Authors:** Yi-Jian Tsai, Sheng-Chieh Huang, Hung-Hsin Lin, Chun-Chi Lin, Yuan-Tzu Lan, Huann-Sheng Wang, Shung-Haur Yang, Jeng-Kai Jiang, Wei-Shone Chen, Tzu-chen Lin, Jen-Kou Lin, Shih-Ching Chang

**Affiliations:** 1 Division of Colon and Rectal Surgery, Department of Surgery, Taipei-Veterans General Hospital, No 201,Sec 2, Shih-Pai Rd, 11217 Taipei, Taiwan; 2 Department of Surgery, Faculty of Medicine, National Yang-Ming University, Taipei, Taiwan

**Keywords:** Colorectal cancer, Gender, Gene mutation

## Abstract

**Background:**

The incidence, site distribution, and mortality rates of patients with colorectal cancer differ according to gender. We investigated gene mutations in colorectal patients and wanted to examine gender-specific differences.

**Methods:**

A total of 1505 patients who underwent surgical intervention for colorectal cancer were recruited from March 2000 to January 2010 at Taipei Veterans’ General Hospital and investigated for gene mutations in K-ras, N-ras, H-ras, BRAF, loss of 18q, APC, p53, SMAD4, TGF-β, PIK3CA, PTEN, FBXW7, AKT1, and MSI.

**Results:**

There were significant differences between male and female patients in terms of tumor location (*p* < 0.0001) and pathological stage (*p* = 0.011). The female patients had significantly more gene mutations in BRAF (6.4 vs. 3.3%, OR 1.985, *p* = 0.006), TGF-β (4.7 vs. 2.5%, OR 1.887, *p* = 0.027), and revealed a MSI-high status (14.0 vs. 8.3%, OR 1.800, *p* = 0.001) than male patients. Male patients had significantly more gene mutations in N-ras (5.1 vs. 2.3%, OR 2.227, *p* = 0.012); however, the significance was maintained only for mutations in BRAF (OR 2.104, *p* = 0.038), MSI-high status (OR 2.003 *p* = 0.001), and N-ras (OR 3.000, *p* = 0.010) after the groups were divided by tumor site.

**Conclusion:**

Gene mutations in BRAF, MSI-high status, and N-ras differ according to gender among patients with colorectal cancer.

## Background

Although the colorectal mucosa and colorectal cancer are morphologically identical in genders, gender-specific differences in incidence, site distribution, and mortality rates of colorectal cancer are evident. These differences were thought to be related with hormonal factors, e.g., estrogen level, or behavioral factors, e.g. nutritional habits, physical activity, and alcohol consumption [1, 2]. Previous studies revealed postmenopausal women treated with estrogen replacement therapy have a significant reduction in both risk and rate of developing colon cancer, [3] and estrogen exposure is a protective factor against microsatellites instability (MSI), while the lack of estrogen in older women increased the risk of MSI-high colon cancer [4]. Alcohol consumption may affect the risk of colorectal cancer and rectal cancer, particularly in men [5].

A recent review on the clinical and molecular characteristics of colon cancer revealed a higher incidence of right-sided colon cancer in women than in men [6, 7]. In addition, high status MSI, CpG island methylator phenotype (CIMP), and BRAF mutations are often observed in right-sided colon cancer [1, 2]. On the other hand, chromosomal instability, which is associated with 60 to 70% cases of colorectal cancer, is more often observed in left sided colon cancer and defective genes include adenomatous polyposis coli (APC), K-ras, deleted in colorectal cancer (DCC), and p53 [8]. Our goal was to identify gender-specific molecular differences, especially current known colorectal-cancer-related gene mutation, in colorectal cancer, and determine its association with the side of tumor distribution.

## Methods

### Study setting and population

This was a retrospective cohort study. Cases were retrieved from the database of the Division of Colorectal Surgery, Taipei Veterans General Hospital. Patients with adenocarcinoma of the colon and rectum, undergoing curative resections, were retrieved. Clinical information that had been prospectively obtained and stored in the database included age, gender, TNM stage, differentiation, location of tumor, pathological prognostic features, personal and family medical history, and follow-up conditions. The right-sided colon was defined as the colon between the cecum and the splenic flexure of the colon. The left sided colon was defined as the colon from the splenic flexure to rectum. Patients who had undergone preoperative chemo-radiotherapy, emergent operations, or who were dead within 30 days of surgery were excluded. In addition, definite germline mutations of MMR genes were noted in 30 patients, and they were also excluded.

### Collection of tumor tissue

All patients gave their informed consent for inclusion before tissue collection before the operation and sample collection. The study was conducted in accordance with the Declaration of Helsinki, and the protocol was approved by the Ethic Committee of Institutional Review Board of Taipei Veterans General Hospital in Taiwan.(no. 2013–04-042B) Tumors were dissected and collected from different quadrants of the tumors and were then snap frozen in liquid nitrogen. Samples were stored at the Taipei Veterans’ General Hospital Biobank.

### DNA isolation and quantification

Samples were obtained from the Biobank for the study. DNA was extracted from the sample using the QIAamp DNA Tissue Kit (Qiagen, Valencia, CA, USA) according to the manufacturer’s protocol. The purity and quantity of DNA were confirmed using Nanodrop ND-1000 Spectrophotometer (Thermo Scientific, Wilmington, DE, USA).

### MassArray-based mutation characterization

According to hotspots found in previous studies and the Catalogue of Somatic Mutations in Cancer (COSMIC) database, The MassDetect colorectal cancer (CRC) panel (v2.0) was designed, enabling the identification of 139 mutations in 12 genes. The primers of polymerase chain reaction (PCR) and extension for the mutations were made with the MassArray Assay Design 3.1 software (Sequenom, San Diego, CA, USA). Multiplexed reactions were spotted onto SpectroCHIP II arrays, DNA fragments were resolved electrophoretically on MassArray Analyzer 4 System (Sequenom, USA), and the spectrum was analyzed (Typer 4.0 software (Sequenom, USA)) to detect mutations.

We defined a putative mutation as one with a 5% abnormal signal and then filtered it by manual review. Sanger sequencing was performed to confirm any detected mutation in *BRAF*, *KRA*S, and *NRAS*. The concordance was 99.1% using MassArray and Sanger sequencing.

### MSI analysis

As international criteria, we used *D5S345*, *D2S123*, *BAT25*, *BAT26*, and *D17S250* as reference microsatellite markers for the determination of MSI, and obtained the primer sequences for these genes from GenBank [9] The detection of MSI was done as previously described. We defined high status MSI as samples with more than or equal to 2 MSI markers, and microsatellite stability as those with 1 or without an MSI marker.

### Statistical analysis

Patient baseline characteristics, including age, gender, tumor site, tumor staging were collected. The patients were divided according to gender. Then, each gene mutation was tested by Pearson’s chi-square test to find the difference between the two groups. All *p* values are two-sided and are considered significant if they are less than .05. The demographic data between two the groups were checked with Pearson chi-square test to identify any possible confounding factors. Data management was done using SPSS software, version 22. (IBM Corp. Armonk, NY, USA).

## Results

From March 2000 to January 2010, 1505 patients were recruited from the database. The demographic data of patients are presented in Table 1.

**Table 1:** Demographic data of patients

			Men	Women
Gender				
Men	990	65.8%		
Women	515	34.2%		
Age (years)				*p* < 0.001
Median(range)	72.17	28~ 107	73.95 (28~ 107)	67.20 (31~ 95)
≤ 70 years	646	42.9%	355 (35.9%)	291 (56.5%)
> 70 years	859	57.1%	635 (64.1%)	224 (43.5%)
Pathological staging				*p* = 0.011
I	212	14.1%	132 (13.3%)	80 (15.5%)
II	564	37.5%	399 (40.3%)	165 (32.0%)
III	473	31.4%	291 (29.4%)	182 (35.3%)
IV	256	17.0%	168 (17.0%)	88 (17.1%)
Tumor localization				*p* < 0.0001
Right colon	400	26.6%	232 (23.4%)	168 (32.6%)
Left colon and rectum	1105	73.4%	758 (76.6%)	347 (67.4%)
Histopathology grade				*p* = 0.368
Well and moderately differentiated	1417	94.2%	936 (94.5%)	481 (93.4%)
Poorly differentiated	88	5.8%	54 (5.5%)	34 (6.6%)

Most of our patients were men (65.8%), and the median age at diagnosis was 72.17 years. The tumors were most often located on left side of the colon and rectum (73.4%), and only 6% of patients had a poorly differentiated histopathological grade. Most patients were at pathological TNM stages II (37.5%) and III (31.4%). The stage IV patients accounted for 17% of all patients. There were significant differences between male and female patients in terms of tumor location (*p* < 0.0001) and pathological stage (*p* = 0.011). For example, the female group had more right-sided tumors, more stage III patients, and less stage II patients. The female patients had significantly higher number of BRAF gene mutations (6.4 vs. 3.3%, OR 1.985, *p* = 0.006), TGF-β mutations (4.7 vs. 2.5%, OR 1.887, *p* = 0.027), and revealed a higher MSI status (14.0 vs. 8.3%, OR 1.800, *p* = 0.001) than male patients. (Table 2).

**Table 2:** Genes with higher mutation rate in female patients

Gene mutation	F vs. M	OR	CI 95%	*p* value
BRAF	6.4 vs. 3.3%	1.985	1.211~3.256	0.006
TGF-β	4.7 vs. 2.5%	1.887	1.066~3.338	0.027
MSI-high status	14.0 vs. 8.3%	1.800	1.286~2.519	0.001

*F* female, *M* male, *OR* odds ratio, *CI* confidence interval

Male patients had significantly higher gene mutations in N-ras (5.1 vs. 2.3%, OR 2.227, *p* = 0.012) than female patients. (Table 3).

**Table 3:** Genes with higher mutation rate in male patients

Gene mutation	M vs. F	OR	CI 95%	*p* value
N-ras	5.1 vs. 2.3%	2.227	1.176~ 4.219	0.012

*M* male, *F* female, *OR* odds ratio, *CI* confidence interval

We then separated the patients according to tumor sides and performed the same statistical analysis. The female patients still had a high number of gene mutations in BRAF (OR 2.104, *p* = 0.038), MSI-high status (OR 2.003 *p* = 0.001) at right side colon, (Table 4), and the male still had a high number of gene mutations in N-ras (OR 3.000, *p* = 0.010) (Table 5).

**Table 4:** Genes with higher mutation rate in female patients after divided by tumor side

Gene mutation	Right side			Left side		
	OR	CI 95%	*p* value	OR	CI 95%	*p* value
BRAF	2.104	1.030~4.298	0.038	1.514	0.739~3.101	0.254
TGF-β	1.897	0.895~4.020	0.091	1.280	0.500~3.280	0.606
MSI-high status	2.003	1.212~3.311	0.006	1.349	0.835~2.179	0.220

*OR* odds ratio, *CI* confidence interval

**Table 5:** Genes with higher mutation rate in male patients after divided by tumor side

Gene mutation	Right side			Left side		
	OR	CI 95%	p value	OR	CI 95%	*p* value
N-ras	1.472	0.541~4.000	0.446	3.000	1.256~7.142	0.010

*OR* odds ratio, *CI* confidence interval

## Discussion

Several studies have revealed that certain genetic and epigenetic differences between sexes may determine colorectal cancer risk. A recent systemic review reported that the proportion of women presenting with right-sided colon cancer, which is often at a more advanced stage at diagnosis, was higher than men [7]. Hendifar et al. reported that in patients with metastatic colorectal cancer, women were more likely to have right-sided or proximal lesion [10]. In our study, the female group was more likely to have right-sided colon cancer and more female patients were at stage III at diagnosis. This is consistent with previous studies.

Our study revealed that there is a gender-specific difference in patients with colorectal cancer regarding gene mutations of BRAF, N-ras, and high status MSI. Sporadic MSI/MMR-deficient (dMMR) colorectal cancer is associated with the BRAF V600E mutation, though its association with CIMP (CpG island methylator phenotype) [11] has been reported. In addition, an earlier study reported that female patients were 8.8 times more likely than male patients to have methylation-positive cancers [12], and previously published studies suggested that [13–15] sporadic MSI/dMMR metastatic colorectal cancer occurred more in female patients. Our study revealed that females had significantly more gene mutations than males in terms of high status MSI and BRAF which has been suggested in previous reports. Breivik et al. reported MSI tumors were more common among old women and younger men [16]. Lindblom reviewed previous study and concluded that estrogen may have a protective effect for MSI cancer in women and a possible mechanism could be an increased methylation. We have also performed further analysis toward gender, age, and MSI status in our study, no significant statistical difference was found in patients below 70 years for MSI tumor by genders.(*p* = 0.334) And the female older than 70 years had 2.45 times risk for MSI tumors than men. (*p* < 0.001).

The approximate frequency of N-ras mutations in colorectal adenocarcinoma is 2 to 8% [17–21]. The frequency in our study was 4.1%, which is consistent with previous studies; however, the significance between genders was not seen in previous reports. While some gender-specific differences were noted in our study, no other chromosomal instability-related genes demonstrated gender-specific differences.

Besides, for the interests in early-onset colorectal cancer (EOCRC) patients, we have done subgroup analysis for EOCRC. EOCRCs are disproportionately located in the distal colon, and there is a longer interval between symptoms and diagnosis [22]. In our study, 63 patients (4.2%) are below 50 years old. Distal distribution was noted (left colon, including rectum, 77%; rectum, 41%), and there was a trend that increased incidence rectal cancer in more men than in women, but there is no statistical significance (*p* = 0.124).

Due to retrospective design and single center data, the study has its inherited limitation. Our databases did not query menopausal status, history of hormone replacement therapy, or contraceptive use; therefore, this limited our ability to investigate its interaction with our findings.

## Conclusions

Gene mutations in high status MSI, BRAF, and N-ras differ according to gender among patients with colorectal cancer. No other chromosomal instability-related genes demonstrated gender-specific differences. Hormone status may play role in the development and pathogenesis of colorectal cancer and warrant further studies to determine it.

## Abbreviations

APC: Adenomatous polyposis coli
CI: Confidence interval
CIMP: CpG island methylator phenotype
CRC: Colorectal cancer
DCC: Deleted in colorectal cancer
MSI: Microsatellite instability
PCR: Polymerase chain reaction
PI3K: Phosphoinositol-3-kinase
TGF-β: Transforming growth factor beta
TNM: Tumor-node-metastasis
